# Evolution of oral health in oral cancer patients with and without dental treatment in place: Before, during and after cancer treatment

**DOI:** 10.4317/jced.54608

**Published:** 2018-02-01

**Authors:** Jesus Nuñez-Aguilar, Ana Fernández-Olavarría, Luis-Guillermo Oliveros-López, Daniel Torres-Lagares, Maria-Angeles Serrera-Figallo, Aida Gutiérrez-Corrales, Jose-Luis Gutiérrez-Pérez

**Affiliations:** 1 PhD, DDS, MSc. University of Seville; 2 DDS, MSc. Oral Surgery Residents. University of Seville; 3 PhD, DDS, MSc. Professor of Oral Surgery. Chairman of Oral Surgery. Department of Stomatology. University of Seville; 4 PhD, DDS, MSc. School of Dentistry. University of Seville; 5 PhD, DMD, Professor of Oral Surgery. Chairman of Oral Surgery. Department of Stomatology. University of Seville

## Abstract

**Background:**

This study aims to evaluate the influence of two dental treatment protocols, outpatient non-regulated treatment versus supervised hospital treatment, on the oral health of patients undergoing oral cancer (only radiochemotherapy treatment, not surgical) treatment.

**Material and Methods:**

The study used a quasi-experimental approach justified on ethical grounds. A total of 41 patients were included in the control group (outpatient non-regulated treatment) and 40 patients in the experimental group (in-hospital supervised treatment). The patients were treated only with chemotherapy (not surgical treatment was made) for oral cancer. This decision was taken by the oncology committee of the hospital without being influenced by this study. Data regarding plaque index, daily brushing habits, appearance of new cavities, need for extractions, appearance of candidiasis and use of prosthetics in both groups were collected at three points throughout the study: before starting cancer treatment, during treatment and after treatment. The values obtained using the Student’s t-test and chi-squared were compared.

**Results:**

Based on similar patient backgrounds, throughout cancer treatment the intervention under study resulted in a decrease in plaque index, necessary extractions, and incidence of decay, as well as an increase in daily brushing among other improvements in oral health observed in the experimental group versus the control group.

**Conclusions:**

From our data, we can confirm that supervised dental treatment performed during oral cancer treatment produced an improvement in the oral health of patients with oral cancer.

** Key words:**Oral cancer, dental treatment, quality of life, oncology, dentistry.

## Introduction

Head and neck carcinomas are the fifth leading cause of cancer in the world’s population, representing 5% of all cancers in men and 2% in women (1). The annual incidence of these carcinomas is 500,000 new cases per year (2,3).

Malignancies in the mucous membrane lining of the oral cavity are epidermoid squamous cell carcinomas (ESCC) in about 90% of cases. Most of these tumors are found in surface areas and could be diagnosed at early stage, but those lesions located in deeper levels usually manifest themselves and are subsequently diagnosed after having grown and reached advanced stages (2).

Alcohol and smoking, and particularly the combination of both, are considered the main etiological risk factors for the development of this malignancy (4-6). Other predisposing etiological factors include infection by the human papillomavirus (7) and the presence of chronic oral inflammation. These two conditions play an important role in patients who have never been smokers or drinkers (8-10). In addition, there are independent risk factors such as poor hygiene and poor oral health that must be considered and more notably present in cancer patients than in healthy subjects (11).

Aggressive treatment of an oncological disease produces inevitable effects on normal cells. Due to its high rate of cell proliferation, the gastrointestinal tract mucosa, including the oral mucosa, is the main place where the toxic effects of cancer treatment are observed (12).

Radiochemotherapy treatment of head and neck tumors affects the entire stomatognathic system, especially the lips, tongue, floor of mouth, oral mucosa, palate, and gums, with the presence of mucositis evident two weeks after starting treatment. In addition, it affects the salivary glands, causing xerostomia; masticatory muscles, resulting in skin fibrosis and muscular atrophy; and dental caries and fungal and bacterial infections, directly affecting the oral health of patients (13-19).

Several studies have indicated the high demand for dental treatment, especially treatment of caries and periodontal disease (57% to 98%) observed in patients diagnosed with oral cancer (20-25). Likewise, many authors recommend dental treatment prior to cancer treatment; extractions are the most frequently performed of these treatments (26,28). Current guidelines usually recommend the removal of any teeth with poor prognosis and a high risk of infection in order to reduce the risk of osteonecrosis in irradiated areas (24).

However, teeth with periodontal disease and pockets of less than 5 mm are likely to be maintained with periodontal treatment (24,25,28). Many of the dental extractions carried out on these patients are not performed due to the impossibility of other treatment, but rather a preference for a more radical approach in cancer patients. Some studies justify this by associating high rates of plaque with an increased risk of ONJ (24). Moreover, further studies are needed to confirm the effectiveness of a radical approach in the treatment of cancer patients via extraction in reducing the risk of osteonecrosis.

Since 1994, authors have recommended that dental therapies be administered prior to cancer treatment in patients with head and neck cancer. Lockhart *et al.* (23) advise that patients receive comprehensive treatment by a dentist in order to prevent complications during and after radiotherapy.

In 2008, Jham *et al.* 2008 published a retrospective study on dental treatment administered to patients prior to radiotherapy, evaluating oral health before, during and after cancer treatment (22). This study does not allow us to analyze the relevance of dental treatment to oral health because there was no control group.

Koga *et al.* included 2,677 patients with head and neck cancer in a retrospective study of a period of ten years, in which the only dental treatments performed were tooth extractions, required by 405 of the patients (15.1%) (29).

In 2014, Saito *et al.* conducted a study of patients with breast cancer that showed lower presence of oral mucositis in patients undergoing dental treatment before and during chemotherapy (30).

The information we have about the relationship between patients’ oral health and dental treatments carried out during oncological treatment is still limited. The aim of this study is to evaluate the impact of a basic regulated dental treatment on the oral health of patients undergoing oral cancer therapy using a quasi-experimental prospective approach, with a view to increasing the published evidence available in this area.

## Material and Methods

A quasi-experimental study was conducted at the Oral and Maxillofacial Surgery Service of the Virgen del Rocio Hospital, in the city of Seville. In mid-2005, the possibility of incorporating some kind of dental treatment into the therapy provided to cancer patients at the same hospital was raised (along the lines of Kielbassa *et al.*) (31).

We received the proposal from the hospital management but needed time to implement the necessary resources, so this study began by including patients with oral cancer (squamous cell carcinoma) in the control group. A total of 41 patients were received from September 2005 to September 2006. After having implemented the resources needed to provide dental treatment, we began treating patients in the experimental group: 40 patients between October 2006 and October 2007.

The inclusion criteria applied in this study were as follows: patients diagnosed with oropharyngeal cancer (squamous cell carcinoma) who had been admitted to the Virgen del Rocio Hospital in Seville and were in need of combined radiochemotherapy cancer treatment. To avoid biases linked to surgical trauma, which is difficult to standardize, only patients who did not undergo surgery were included. Other inclusion criteria were non-edentulous patients, and patients with a Karnofsky index of equal to or greater than 50%.

The exclusion criteria applied in this study were as follows: patients who voluntarily refused to the treatment proposed by the specialists of our service and opted for another treatment alternative to what oncologists had recommended; patients who were referred to another hospital; patients who chose not to be treated for their disease; patients who willingly ceased cancer treatment; patients who refused to submit to any part of the study or refused to consent to the scientific use of their data; failure to sign or breach of informed consent; patients who died during the study.

All patients were treated by Integral Consultation Service for Oropharyngeal Tumors at the Virgen del Rocio Hospital in Seville; once tumor evolution had been assessed, patients were scheduled to undergo cancer treatment. At that time, patients’ current dental health, habits and oral problems were assessed. Patients in the control group were informed and advised of the care they should receive during radiochemotherapy treatment. This dental treatment was established and monitored at primary care level centers ( Table 1). Experimental group patients underwent supervised dental treatment, following Kielbassa *et al.’s* guidelines (31) ( Table 1), along with their radiochemotherapy treatment, but this treatment was held in the facilities of the hospital.

**Table 1:**
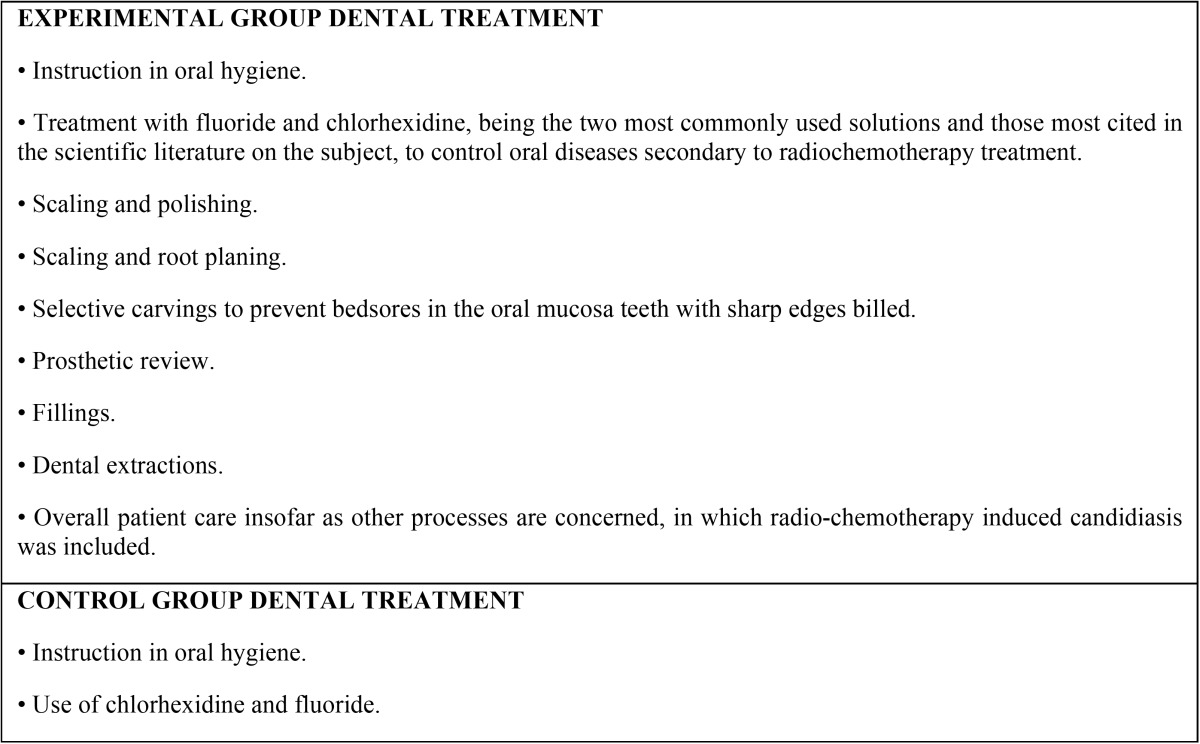
List of in-hospital treatments performed on patients in each group.

The study protocol was approved by the Ethics Committee of the Virgen del Rocio Hospital. All patients read and signed their informed consent to participate in the study. The guidelines for human experimentation outlined in the Declaration of Helsinki were also carefully followed.

Observers collected all data on the oral health of patients in both the control and experimental groups. A simple odontogram was used for this purpose. Data were as follows: clinical decay, whether the patient had an oral prosthesis, teeth that required dental extraction during the study and the reason for the latter. The plaque index was measured using plaque disclosing tablets (Plac-control®, Dentaid, Spain) and by obtaining the result of dividing the number of surfaces with plaque by the total number of surfaces, multiplied by one hundred. Likewise, the number of daily brushings was recorded, as well as whether or not the patient presented with oral candidiasis. In each group, data collection was performed prior to (one month before the radiochemotherapy), during (after completion of 60% of radiochemotherapy treatments) and after cancer treatment (twelve months after beginning the study).

The collected data were encoded into a data file in SPSS v.11 (IBM, USA) for statistical analysis. The descriptive study was conducted using mean and standard deviation or percentage, depending on the type of variable. To identify differences between the two groups that could be statistically significant, the chi-squared test or Student’s t-test method was applied, according to the variable being compared between the two groups.

Similarly, variables were recorded during the three data collection periods of the study (before, during and after cancer treatment) in both the control and experimental groups in order to analyze the difference between these points, identifying the growth or decline of each throughout the study. These data also were compared between the two groups.

## Results

First, indicated treatments were performed on each study group at three specific times, before treatment, during and after cancer treatment ( Table 2). Data relating to oral health at each of these three stages were also collected ( Table 3). Developments over this period were analyzed by comparing each of the variables in each group ( Table 4).

**Table 2:**
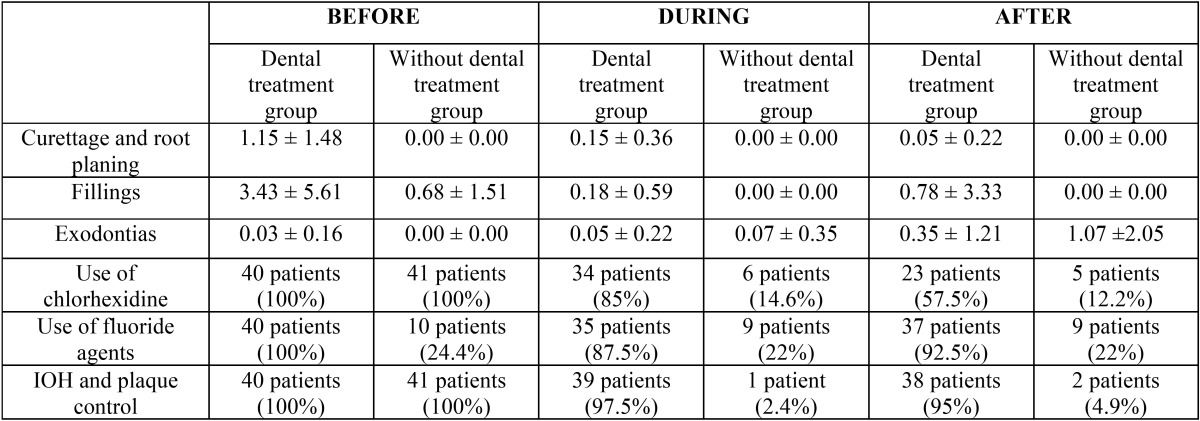
Treatments applied to the groups before, during and after treatment with radiochemotherapy. The data from the experimental group gives an idea of in-hospital therapeutic efforts. For the control group, treatments were performed on an unsupervised outpatient basis, and data were taken from questions asked of patients in the group during study follow-up visits. The pairs of values at each time of the study that showed significant differences between the two groups (*p* < 0.05, chi-squared and Student’s t-test) are indicated in bold.

**Table 3:**
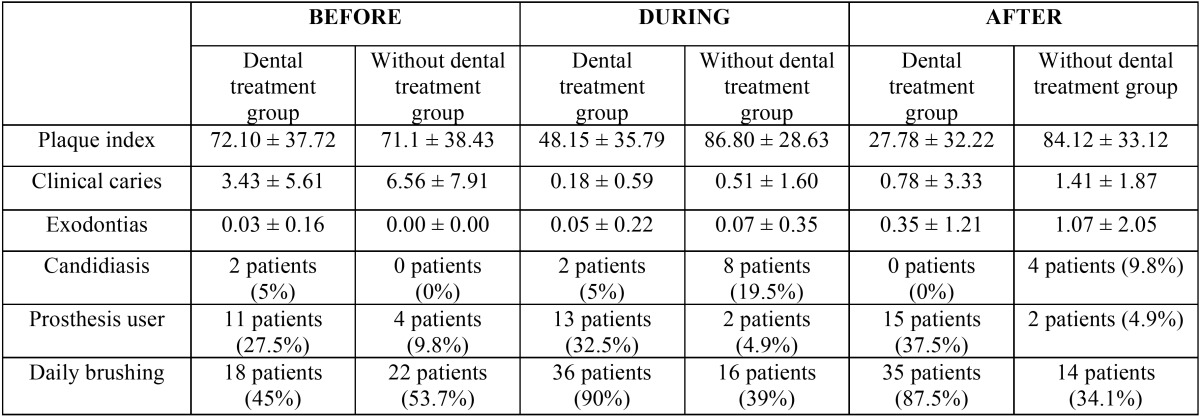
Data on oral health status in each group before, during and after radiochemotherapy. The pairs of values at each time of the study that showed significant differences between the two groups (*p* < 0.05, chi-squared and Student’s t test) are indicated in bold.

**Table 4:**
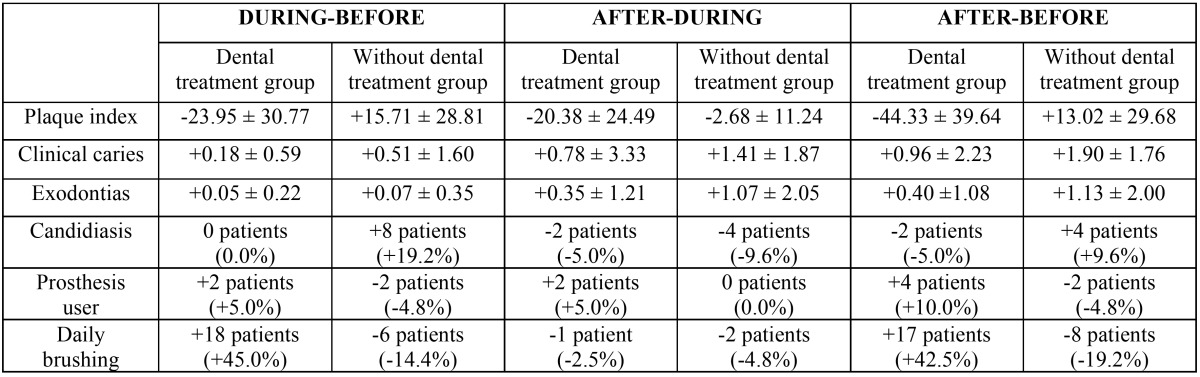
Evolution of oral health status in both groups over different periods of cancer treatment. The pairs of values at each time of the study that showed significant differences between the two groups (*p* < 0.05, chi-squared and Student’s t) are indicated in bold.

**Figure 1:**
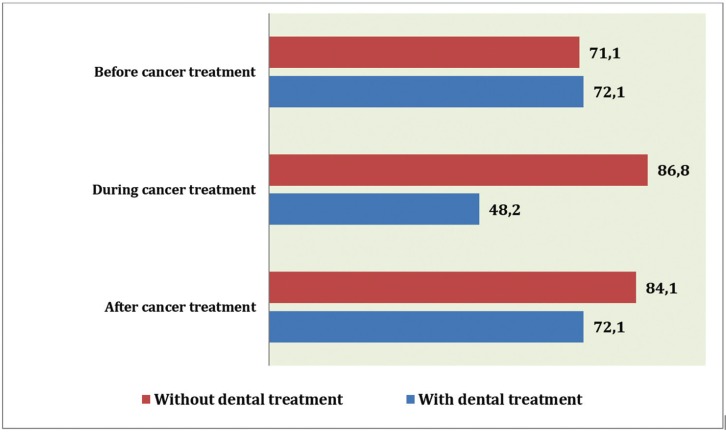
Plaque index in groups at different periods of the study (%).

Beyond the absolute data, we will focus mainly on the analysis of data evolution. However, it is interesting to note plaque index scores and their evolution in both study groups ( Table 3,  Table 4, Fig. 1). We can observe how such a basic indicator of oral health, with so many implications in the prevention of oral diseases, behaves differently. The intervention proved able to control this indicator during cancer treatment, ending at similar levels to those observed pre-treatment. In the control group, the plaque index soars and remains high throughout treatment, with all of the clinical implications that this entails.

Upon focusing on the data increases/decreases of variables, and more specifically by comparing variables before and during cancer treatment, we can state that in the experimental (with dental treatment) group, plaque index was reduced by -23.95 ± 30.77. On the other hand, the control (without dental treatment) group saw an increase in plaque index of ± 28.81 +15.71 between these two periods; the difference was statistically significant (*p* < 0.0001). The incidence of new caries in the dental treatment group was 0.18 ± 0.59. In the control group, this value was 0.51 ± 1.60, finding no statistically significant differences.

Furthermore, during this period there was no difference between the number of tooth extractions performed in both groups (experimental: 0.05 ± 0.22; control: 0.07 ± 0.35). Two patients in the experimental group began using prostheses while undergoing treatment, while in the group without dental treatment, two patients stopped using them. The number of daily brushings increased by 18 patients in the experimental group, while it decreased by six patients in the control group (*p* > 0.0001). The number of patients with candidiasis increased in the control group (+8 patients) but remained stable in the experimental group (*p* < 0.001).

With regard to the values found upon comparing the post-treatment period and the intermediate point, we can see that the plaque index was reduced in the experimental group by -20.38 ± 24.49. The reduction in plaque index in the control group was lower: -2.68 ± 11.24 (*p* < 0.001). The number of clinical caries at the end of cancer treatment compared to the number during treatment had increased by 0.78 ± 3.33 for the experimental group and 1.41 ± 1.87 for the control group (*p* < 0.01). The number of extractions performed in both groups also showed significant differences between these periods (experimental group: 0.35 ± 1.21; control group: 1.07 ± 0.50; *p* < 0.05).

Regarding the use of prostheses, there were no changes observed in the control group during this period. However, two patients in the experimental group began using prostheses. This was not a statistically significant difference. Regarding daily brushing habits, one of the patients in the experimental group and two in the control group ceased brushing, but this was not a statistically significant difference. The presence of candidiasis decreased in two patients in the experimental group and four patients in the control group throughout this period (no significant difference).

Finally, with regard to the evolution of variables measured throughout the cancer treatment, we can state that in patients undergoing dental treatment, plaque index decreased by 44.33 ± 39.64. In patients without dental treatment, plaque index increased by 13.02 ± 29.68 (*p* < 0.0001). The number of clinical caries in the experimental group was +0.96 ± 2.23, while the control group showed +1.90 ± 1.76 (*p* < 0.01).

The number of dental extractions was lower in patients with treatment (0.40 ± 1.08) than in patients in the control group (1.13 ± 2.00) (*p* < 0.05). Throughout the cancer treatment, 10% of patients (four patients) in the experimental group began using prosthetics, while two patients (-4.8%) in the control group had to stop using them (*p* < 0.05). Seventeen patients in the experimental group increased their daily brushing, while eight patients in the control group decreased their daily brushing (*p* < 0.0001). The presence of candidiasis during cancer treatment decreased in two patients in the experimental group and increased in four patients in the control group (*p* < 0.05).

## Discussion

Multiple studies have detailed the enormous amount of side effects that appear in the mouth after radio and/or chemotherapy treatments for head and neck tumors, explaining how these bucco-dental disorders adversely affect patients (5.12 to 16.18, 25,27,28). It is evident that dentists are crucial to the early detection of oral cancer, but their role should not end there. (17,20-24,26,28-30,32-39)

In 1994, Lockhart *et al.* (23) created dental treatment protocols for use prior to cancer treatment in patients with head and neck cancer that help avoid the complications of radiotherapy.

In 2013, Niewald *et al.* (35) designed a retrospective study of 90 patients that evaluated the dental health of patients before undergoing radiotherapy and its possible involvement as a risk factor for the development of mandibular osteoradionecrosis. They concluded that an increase in monitoring and dental treatment would result in fewer problems for patients in the future.

Barrios *et al.* (19) conducted a study in 2015 of 142 patients in which they assessed the relationship between oral health and quality of life in patients suffering from oral cancer. A statistically significant difference was seen in patients who received dental treatment; a better outcome was observed in these patients, a finding corroborated our study.

Upon reviewing the results obtained in the control group, the present reveals statistically significant differences in the improvement of overall oral health of patients with a primary care protocol. It should be noted that no dental treatments were performed in the control group, but we devised a patient follow-up protocol with corresponding administration of toothpaste, mouthwashes and oral hygiene instruction. Although treatment in this group was limited to advice and instruction in oral hygiene practices, these were shown to benefit patients.

In our study, prior to radiochemotherapy treatment for cancer, both the control and experimental groups had similar characteristics and treatment needs. During radiochemotherapy, this situation changed dramatically. The most popular treatments (almost entirely in the experimental group) were scaling and polishing, scaling and root planing, and instruction in oral hygiene and plaque control. The change in the attitudes and oral health habits of the experimental group was significant, with 95.12% of patients voluntarily attending hygiene education sessions (*p* < 0.001). One possible reason for this was that they received their dental treatment at the same place where the other professionals who treated their cancer were.

This improvement in oral care leads to a real improvement in the oral health of the patient, as was observed in the experimental group, with 36 patients who brushed daily during cancer treatment, compared to 16 patients who brushed in the control group. With all controls, an improved better plaque index is to be expected, and the difference between the experimental and control groups almost double. We also found a small number of new clinical caries in the experimental group compared to the control group.

The experimental group included more patients who used their prosthesis during radiochemotherapy than in the control group (a ratio of 13 patients out of 40 versus a ratio of 2 out of 41 (*p* < 0.001). Patients with poor oral health who were undergoing cancer treatments were able to maintain and properly use prostheses during radiochemotherapy. These patients saw a remarkable improvement in their dental health and the optimal state of the oral mucosa, which can support the loads of prostheses.

The data obtained from both groups during the post-radiochemotherapy treatment pointed to the positive influence of regulated dental check-ups in controlling the oral health of these patients. In the control group, the plaque index increased significantly, patients brushed less, and the number of patients who carried dentures after radiochemotherapy decreased. In contrast, in the experimental group, plaque index decreased from 71.32% before treatment to 27.09% (*p* < 0.001). These patients brushed much better after treatment compared to the periods before and during radiochemotherapy, going from 18 patients who did not brush to a total of 35 patients who brushed in the experimental group, whereas the control group saw a decrease in the number of brushes (*p* < 0.001). All patients who used prostheses at baseline in the experimental group continued using them after radiochemotherapy. This data seems very relevant due to its high clinical significance.

Our study is relevant because it enables us to evaluate the importance of dental treatment in the oral health of patients with oral cancer who are undergoing cancer therapies, divided into two study groups. The literature lacks publications that prospectively observe patients’ oral health throughout cancer treatment (before, during and after).

The typical dental treatment carried out on these patients involved dental extractions. In this paper, we propose conservative dental therapies that avoid radical treatment that would directly affect the quality of life of patients, in keeping with the guidelines published by Kielbassa *et al.* (31).

Proper oral hygiene control and supervised monitoring improved control of plaque in the experimental group, whereas these results worsened in the control group. The association between these hygiene sessions, use of fluoride agents, brushing and use of chlorhexidine resulted in fewer new caries observed in the experimental group when compared with the control group.

Jham *et al.* also studied the evolution in oral health of patients before, during and after cancer therapy and found similar results. (22) They performed regulated dental treatment prior to radiotherapy that preserved restorable teeth, with only 50% of patients requiring extractions. However, they do not compare the results to a control group, which precludes analysis of the importance of dental treatment in oral health, including its effect on the appearance of new cavities and mucositis.

Saito *et al.* (30) observed a decrease in complications in the oral cavity after chemotherapy, mainly in the reduced appearance of mucositis in patients who attended dental treatment sessions based on hygiene and periodontal treatment. The influence of this treatment cannot be individually assessed because these hygienic measures were also taught to the control group of patients with oral mucositis.

Bertl *et al.* (32) analyzed the overall oral health of patients with head and neck cancer, as well as the dental care they received. Only 52% of patients requested dental check-ups before cancer therapy, although it was recommended to all of them, and 80% of them needed dental treatment.

This leads us to believe that there is a lack of knowledge about the importance of good oral health and prevention of radiochemotherapy complications. Professionals are responsible for providing patients with this information and establishing protocols of oral hygiene and preventive measures that are accessible to patients.

Oral cancer patients should be treated in a multidisciplinary hospital, in an area where the dentist has complete communication with the rest of the team involved in treating the pathology (nurses, nutritionists, psychologists, oncologists, and oral and maxillofacial surgeons).

To summarize the results of our study, as presented and discussed above, we can state that the first part of cancer treatment saw an increase in daily brushing and a strong decrease in the rate of plaque in the experimental versus control groups. The second part of cancer treatment saw a decreased plaque index, number of new cavities and number of extractions in the experimental group. This demonstrates that implementing a protocol of formal dental control care in hospitals during cancer treatment, as well as integrating it into further cancer treatment and outpatient unregulated treatment, leads to a decrease in plaque index, number of extractions needed and incidence of decay, as well as an increase in daily brushing and other improvements to overall oral health.
